# Supplementary material for: Changes in planned and unplanned canopy openings are linked in Europe’s forests
Source: Nat Commun. 2024 Jun 4;15:4741. doi: 10.1038/s41467-024-49116-0 (PMC11150470; doi:10.1038/s41467-024-49116-0)
Supplement: Supplementary file 1 — Supplementary Information [file 41467_2024_49116_MOESM1_ESM.pdf]

Changes in planned and unplanned canopy openings are linked in Europe's forests

Rupert Seidl, Cornelius Senf

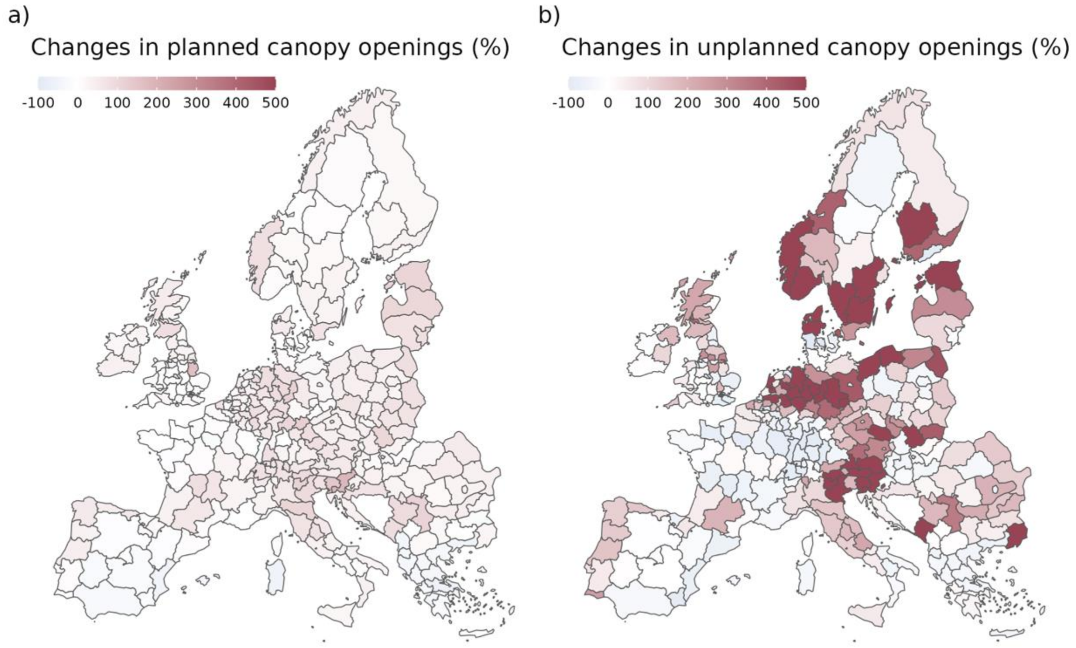

Supplementary Fig. 1 Spatial variation in the change of (a) planned and (b) unplanned canopy openings in Europe's forests 1986-2020. Spatial units are NUTS2, and changes contrast the early 21st century to the late 20th century. Please note that values are capped at 500% for the sake of visualization, for the full range of data see Fig. 2a. Administrative boundaries © EuroGeographics

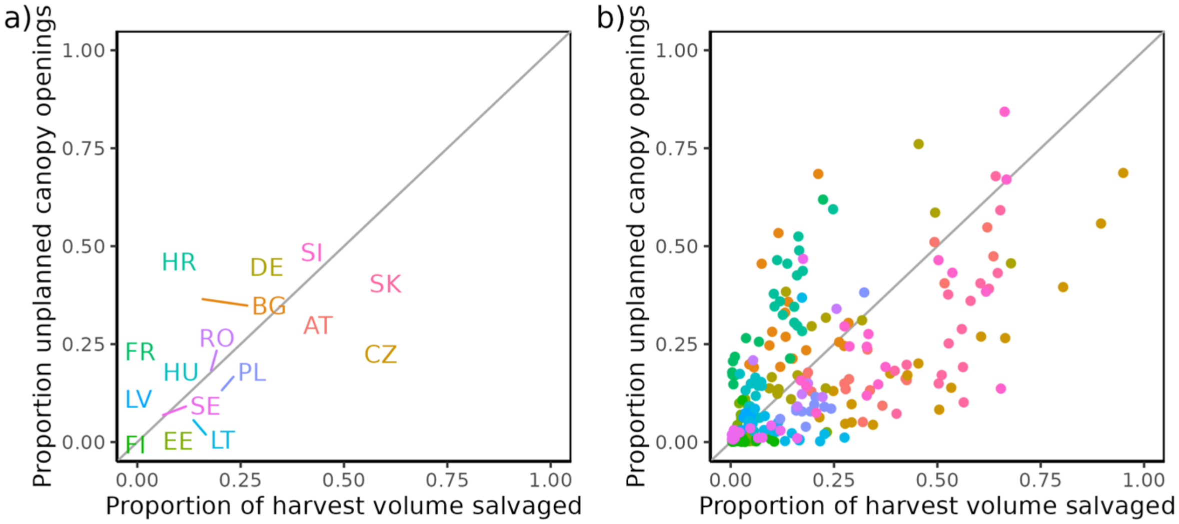

Supplementary Fig. 2: Comparison of the proportion of timber volume salvage harvested following disturbance by ecological agents (x-axis, independent data from [42]) to the proportion of unplanned canopy openings (y-axis, this study) at aggregated national (a) and annual national (b) levels. Data was available for 16 European countries: AT = Austria, BG = Bulgaria, CZ = Czechia, DE = Germany, EE = Estonia, FI = Finland, FR = France, HR = Croatia, HU = Hungary, LT = Lithuania, LV = Latvia, PL = Poland, RO = Romania, SE = Sweden, SI = Slovenia and SK = Slovakia.

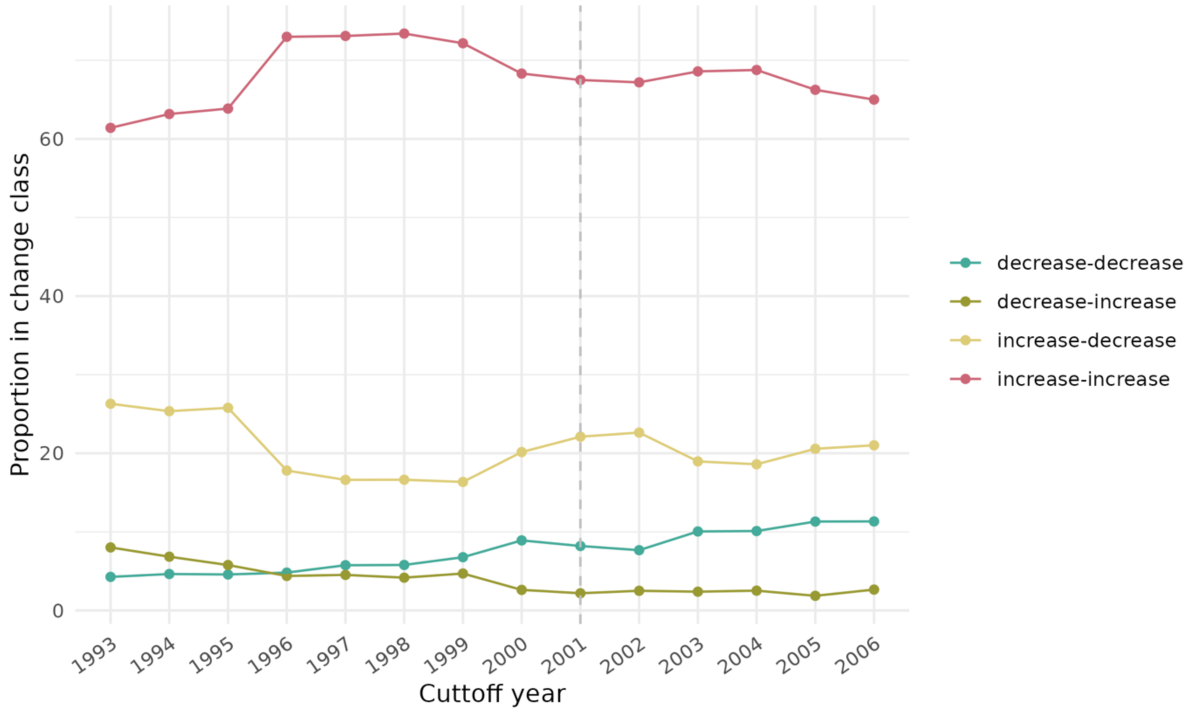

Supplementary Fig. 3 Sensitivity of the four change classes (see Fig. 2) to variable cutoff years used to separate the time series into two periods. The vertical line indicates the cutoff year used for the analyses presented in the main text, separating the data into the late 20th century (1986 – 2000) and the early 21st century (2001 – 2020).
