# Supplementary material for: Changes in planned and unplanned canopy openings are linked in Europe’s forests
Source: Nat Commun. 2024 Jun 4;15:4741. doi: 10.1038/s41467-024-49116-0 (PMC11150470; doi:10.1038/s41467-024-49116-0)
Supplement: Supplementary file 3 — Reporting Summary [file 41467_2024_49116_MOESM3_ESM.pdf]

nature portfolio

Corresponding author(s): Rupert Seidl

Last updated by author(s): May 3, 2024

Reporting Summary

Nature Portfolio wishes to improve the reproducibility of the work that we publish. This form provides structure for consistency and transparency in reporting. For further information on Nature Portfolio policies, see our Editorial Policies and the Editorial Policy Checklist.

Statistics

For all statistical analyses, confirm that the following items are present in the figure legend, table legend, main text, or Methods section.

n/a	Confirmed	
☐	☒	The exact sample size (*n*) for each experimental group/condition, given as a discrete number and unit of measurement
☐	☒	A statement on whether measurements were taken from distinct samples or whether the same sample was measured repeatedly
☐	☒	The statistical test(s) used AND whether they are one- or two-sided<br>*Only common tests should be described solely by name; describe more complex techniques in the Methods section.*
☒	☐	A description of all covariates tested
☐	☒	A description of any assumptions or corrections, such as tests of normality and adjustment for multiple comparisons
☐	☒	A full description of the statistical parameters including central tendency (e.g. means) or other basic estimates (e.g. regression coefficient) AND variation (e.g. standard deviation) or associated estimates of uncertainty (e.g. confidence intervals)
☐	☒	For null hypothesis testing, the test statistic (e.g. *F*, *t*, *r*) with confidence intervals, effect sizes, degrees of freedom and *P* value noted<br>*Give P values as exact values whenever suitable.*
☒	☐	For Bayesian analysis, information on the choice of priors and Markov chain Monte Carlo settings
☒	☐	For hierarchical and complex designs, identification of the appropriate level for tests and full reporting of outcomes
☐	☒	Estimates of effect sizes (e.g. Cohen's *d*, Pearson's *r*), indicating how they were calculated

Our web collection on statistics for biologists contains articles on many of the points above.

Software and code

Policy information about availability of computer code

Data collection	Data processing code is available under https://doi.org/10.5281/zenodo.11070255
Data analysis	Data analysis code is available under https://doi.org/10.5281/zenodo.11070255

For manuscripts utilizing custom algorithms or software that are central to the research but not yet described in published literature, software must be made available to editors and reviewers. We strongly encourage code deposition in a community repository (e.g. GitHub). See the Nature Portfolio guidelines for submitting code & software for further information.

Data

Policy information about availability of data

All manuscripts must include a data availability statement. This statement should provide the following information, where applicable:

- Accession codes, unique identifiers, or web links for publicly available datasets
- A description of any restrictions on data availability
- For clinical datasets or third party data, please ensure that the statement adheres to our policy

The data of the European disturbance map Version 1.1.2 is deposited on Zenodo under accession code: https://doi.org/10.5281/zenodo.3924380.
The data used for the initial disturbance attribution algorithm is deposited on Zenodo under accession code: https://zenodo.org/records/8202241.
The data from the FORWIND database is deposited on figshare under accession code: https://doi.org/10.6084/m9.figshare.9555008.

All original data of this study are deposited on Zenodo under accession code: https://doi.org/10.5281/zenodo.11070255.

Research involving human participants, their data, or biological material

Policy information about studies with human participants or human data. See also policy information about sex, gender (identity/presentation), and sexual orientation and race, ethnicity and racism.

Reporting on sex and gender	N/A
Reporting on race, ethnicity, or other socially relevant groupings	N/A
Population characteristics	N/A
Recruitment	N/A
Ethics oversight	N/A

Note that full information on the approval of the study protocol must also be provided in the manuscript.

Field-specific reporting

Please select the one below that is the best fit for your research. If you are not sure, read the appropriate sections before making your selection.

☐ Life sciences ☐ Behavioural & social sciences ☒ Ecological, evolutionary & environmental sciences

For a reference copy of the document with all sections, see nature.com/documents/nr-reporting-summary-flat.pdf

Ecological, evolutionary & environmental sciences study design

All studies must disclose on these points even when the disclosure is negative.

Study description	Analysis of all disturbance patches in Europe's forests, identified from remote sensing, attributed to planned and unplanned canopy openings.
Research sample	All disturbance patches in Europe's forests, i.e., a total of 41.1 Mill. ha of forest area
Sampling strategy	No sampling for disturbance patches, analysis of the complete population of disturbance patches; for reference data for attribution, locations for planned and unplanned canopy openings were sampled from peer-reviewed papers, published databases, and in recent hotspots of disturbance
Data collection	Disturbance data was collected by Landsat satellites; reference data for attribution was compiled from peer-reviewed papers, published databases, and in recent hotspots of disturbance
Timing and spatial scale	Start: 1986, end: 2020, periodicity: annual, spatial extent: Europe, spatial grain: 30m
Data exclusions	No data were excluded from the analysis
Reproducibility	Code and analyses are provided to ensure reproducibility
Randomization	Not applicable
Blinding	Not applicable

Did the study involve field work? ☐ Yes ☒ No

Reporting for specific materials, systems and methods

We require information from authors about some types of materials, experimental systems and methods used in many studies. Here, indicate whether each material, system or method listed is relevant to your study. If you are not sure if a list item applies to your research, read the appropriate section before selecting a response.

Materials & experimental systems

n/a	Involved in the study
☒	☐ Antibodies
☒	☐ Eukaryotic cell lines
☒	☐ Palaeontology and archaeology
☒	☐ Animals and other organisms
☒	☐ Clinical data
☒	☐ Dual use research of concern
☒	☐ Plants

Methods

n/a	Involved in the study
☒	☐ ChIP-seq
☒	☐ Flow cytometry
☒	☐ MRI-based neuroimaging

Plants

Seed stocks: Not applicable

Novel plant genotypes: Not applicable

Authentication: Not applicable
